# Bulimic behavior in euthymic bipolar disorder patients

**DOI:** 10.1192/j.eurpsy.2025.1384

**Published:** 2025-08-26

**Authors:** M. Abdelkefi, S. Ellouze, R. Jbir, W. Abid, N. Bouattour, N. Halouani, M. Turki, J. Aloulou

**Affiliations:** 1 Psychiatry B, Hedi Chaker university hospital, Sfax, Tunisia

## Abstract

**Introduction:**

Bulimic behavior has been increasingly recognized in patients with bipolar disorder (BD). Even during euthymic phases, individuals with BD may remain vulnerable to disordered eating patterns such as bulimia and binge eating.

**Objectives:**

This study aims to examine the occurrence of bulimic behavior in euthymic patients with BD and identify associated clinical and sociodemographic factors.

**Methods:**

We conducted a cross-sectional, descriptive, and analytical study of 93 patients followed for bipolar disorder at the psychiatry outpatient unit at the Hedi Chaker University Hospital in Sfax. The questionnaire included sociodemographic data, medical and psychiatric history, and anthropometric characteristics. Bulimic behavior was assessed using the Bulimic Investigatory Test Edinburgh (BITE).

**Results:**

The mean age of the participants was 41.49±12.33 years, with a M/F sex ratio of 2.58. Among the patients, 58.1% were married, 45.2% had secondary education, and 47.3% were unemployed. Personal somatic history was reported by 35.5%, while 11.8% had psychiatric comorbidities in addition to bipolar disorder.

The mean body mass index (BMI) was 27.4 kg/m² (SD=5.96), with 29% of patients being overweight and 31.2% classified as obese.

Eight patients (8.6%) had BITE scores above the threshold of 20, indicating bulimic behavior.

Significant associations were found between elevated BITE scores and female gender (p=0.012), comorbid medical conditions (p=0.005), family history of schizophrenia (p=0.024), weight (p<10⁻³), BMI (p<10⁻³), hypomanic residual symptoms (p<10⁻³), irregular follow-up (p=0.027), and delayed management of BD (p=0.04).

**Conclusions:**

Our results highlight the importance of early identification and comprehensive management of disordered eating in bipolar patients, even during periods of mood stability, to optimize overall health and psychiatric outcomes.

**Disclosure of Interest:**

None Declared

